# Haploinsufficiency of *SF3B2* causes craniofacial microsomia

**DOI:** 10.1038/s41467-021-24852-9

**Published:** 2021-08-03

**Authors:** Andrew T. Timberlake, Casey Griffin, Carrie L. Heike, Anne V. Hing, Michael L. Cunningham, David Chitayat, Mark R. Davis, Soghra J. Doust, Amelia F. Drake, Milagros M. Duenas-Roque, Jack Goldblatt, Jonas A. Gustafson, Paula Hurtado-Villa, Alexis Johns, Natalya Karp, Nigel G. Laing, Leanne Magee, University of Washington Center for Mendelian Genomics, Sureni V. Mullegama, Harry Pachajoa, Gloria L. Porras-Hurtado, Rhonda E. Schnur, Jennie Slee, Steven L. Singer, David A. Staffenberg, Andrew E. Timms, Cheryl A. Wise, Ignacio Zarante, Jean-Pierre Saint-Jeannet, Daniela V. Luquetti

**Affiliations:** 1 Hansjorg Wyss Department of Plastic and Reconstructive Surgery, NYU Langone Medical Center, New York, NY USA; 2 Department of Molecular Pathobiology, New York University College of Dentistry, New York, NY USA; 3 Department of Pediatrics, Division of Craniofacial Medicine, University of Washington, Seattle, WA USA; 4 Center for Developmental Biology and Regenerative Medicine, Seattle Children’s Research Institute, Seattle, WA USA; 5 Division of Clinical and Metabolic Genetics, Department of Pediatrics, The Hospital for Sick Children, University of Toronto, Toronto, ON Canada; 6 The Prenatal Diagnosis and Medical Genetics Program, Department of Obstetrics and Gynecology, Mount Sinai Hospital, University of Toronto, Toronto, ON Canada; 7 Department of Diagnostic Genomics, Path West Laboratory Medicine, QEII Medical Centre, Hospital Avenue, Nedlands, WA Australia; 8 Genetics Program, Peterborough Regional Health Centre, Peterborough, ON Canada; 9 Department of Otolaryngology/Head and Neck Surgery, University of North Carolina, Chapel Hill, NC USA; 10 Hospital Edgardo Rebagliati Martins, EsSalud, Lima Peru; 11 Genetic Services of Western Australia, King Edward Memorial Hospital, Perth, WA Australia; 12 Pontificia Universidad Javeriana and Centro Médico Imbanaco, Cali, Colombia; 13 Division of Plastic and Maxillofacial Surgery, Children’s Hospital Los Angeles, Los Angeles, CA USA; 14 Department of Pediatrics, London Health Sciences Centre, Division of Medical Genetics, Western University, London, ON Canada; 15 Neurogenetic Diseases Group, Harry Perkins Institute of Medical Research and Centre for Medical Research, University of Western Australia, Nedlands, WA Australia; 16 Division of Plastic and Reconstructive Surgery, Children’s Hospital of Philadelphia, Philadelphia, PA USA; 17 GeneDx, Gaithersburg, MD USA; 18 Universidad Icesi and Fundacion Clinica Valle del Lili, Cali, Colombia; 19 Clinica Comfamiliar Risaralda, Pereira, Colombia; 20 Dept of Pediatrics, Cooper Medical School of Rowan University; Division of Genetics, Cooper University Health Care, Camden, NJ USA; 21 Perth Children’s Hospital, Nedlands, WA Australia; 22 Human Genomics Institute, Pontificia Universidad Javeriana, Bogotá, Colombia; 23 Hospital Universitario San Ignacio, Bogotá, Colombia

**Keywords:** Genome informatics, Medical genomics, DNA sequencing

## Abstract

Craniofacial microsomia (CFM) is the second most common congenital facial anomaly, yet its genetic etiology remains unknown. We perform whole-exome or genome sequencing of 146 kindreds with sporadic (n = 138) or familial (n = 8) CFM, identifying a highly significant burden of loss of function variants in *SF3B2* (P = 3.8 × 10^−10^), a component of the U2 small nuclear ribonucleoprotein complex, in probands. We describe twenty individuals from seven kindreds harboring de novo or transmitted haploinsufficient variants in *SF3B2*. Probands display mandibular hypoplasia, microtia, facial and preauricular tags, epibulbar dermoids, lateral oral clefts in addition to skeletal and cardiac abnormalities. Targeted morpholino knockdown of *SF3B2* in *Xenopus* results in disruption of cranial neural crest precursor formation and subsequent craniofacial cartilage defects, supporting a link between spliceosome mutations and impaired neural crest development in congenital craniofacial disease. The results establish haploinsufficient variants in *SF3B2* as the most prevalent genetic cause of CFM, explaining ~3% of sporadic and ~25% of familial cases.

## Abstract (web summary)

Despite being a common congenital facial anomaly, the genetic etiology of craniofacial microsomia (CFM) is not well understood. Here, the authors use exome and genome sequencing of 146 individuals with CFM to identify haploinsufficient variants in SF3B2 as a prevalent underlying cause.

## Introduction

Craniofacial microsomia (CFM, MIM#164210), also termed hemifacial microsomia, oculo-auricular-vertebral spectrum (OAVS) or Goldenhar syndrome, comprises a variable phenotype, with the most common features including auricular malformations and underdevelopment of the mandible on one or both sides. Other frequently affected tissues include the middle ear ossicles, temporal bone, zygoma, and cranial nerves. Microtia in the absence of other anomalies is believed to represent the mildest form of CFM^1,2^. At the other end of the phenotypic spectrum, CFM can be associated with multiple anomalies including lateral oral clefts, epibulbar dermoids, and anomalies of the nervous, vertebral, renal, and cardiac systems. Multiplex kindreds with presumably dominant inheritance of CFM demonstrate the wide phenotypic variability of this condition^3,4^.

Previous studies have demonstrated that this pattern of malformations is a condition of etiologic heterogeneity, with genetic and non-genetic risk factors^1,5,6^. Genes involved in embryologic formation of the ears and mandible involve a variety of cellular processes. Variants in transcription factors involved in neural crest cell migration and patterning (TFAP2A, SIX1, SIX5, EYA1, HOXA10, HOXA2), chromatin modifiers (CHD7, KMT2D, KDM6A), growth factors and their receptors (GDF6, FGF3, FGF10, FGFR2, FGFR3), DNA pre-replication complexes (ORC1, ORC4, ORC6, CDC6, CDT1), ribosome assembly (TCOF1, POL1RC, POL1RD), and the spliceosome (EFTUD2, TXNL4A, SF3B4) have been implicated in monogenic syndromes that include malformation of the ears or mandible^7^. Variants in *MYT1* have been identified in unrelated individuals with CFM, suggesting a possible role in disease pathogenesis^8,9^. The two largest families described in the literature to date demonstrating dominant inheritance of CFM identified linkage of the trait to chromosomal bands 11q12-13 and 14q32; however, the responsible gene within each linkage interval was not identified at the time of study^10,11^. Despite these advances, the role of rare variants with large effect on disease risk remains unknown. Here, we show a role for haploinsufficient variants in *SF3B2* as the most frequent genetic cause of CFM identified to date.

## Results

### Exome sequencing of CFM kindreds

**Fig. 1:**
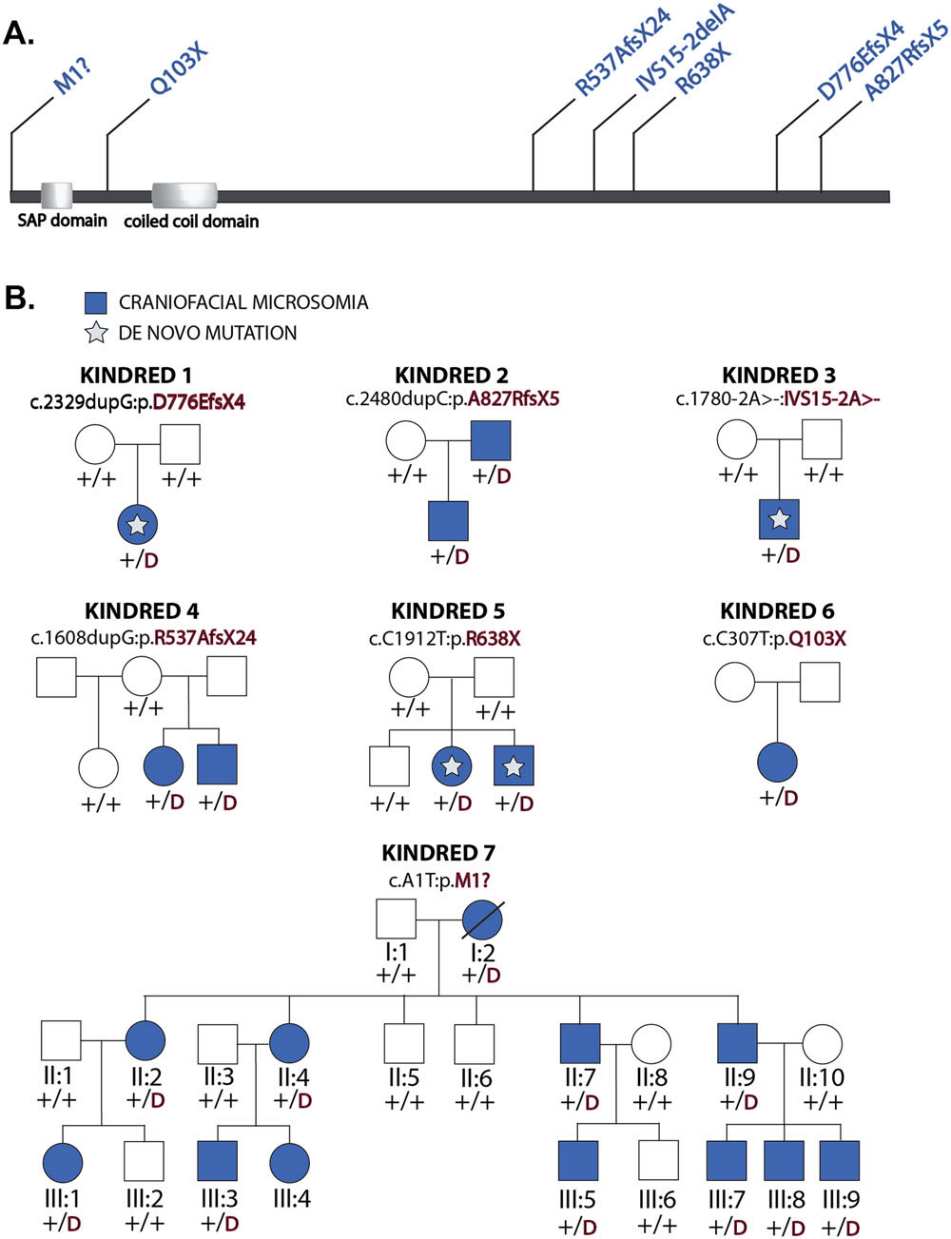
*SF3B2* variants in pedigrees with craniofacial microsomia. **A`** Domain structure of SF3B2 with variants identified noted above. Domain annotation based on UniProt accession Q13435. **B** Pedigrees of kindreds with *SF3B2* LOF variants. De novo or transmitted LOF variants are indicated above each pedigree, with stars denoting confirmed de novo variants. ‘+’ represents a wild type allele, and ‘D’ represents the LOF variant in *SF3B2* indicated above the pedigree. Individuals with no genotype listed represent those in whom genomic DNA was not available for study.

**Fig. 2:**
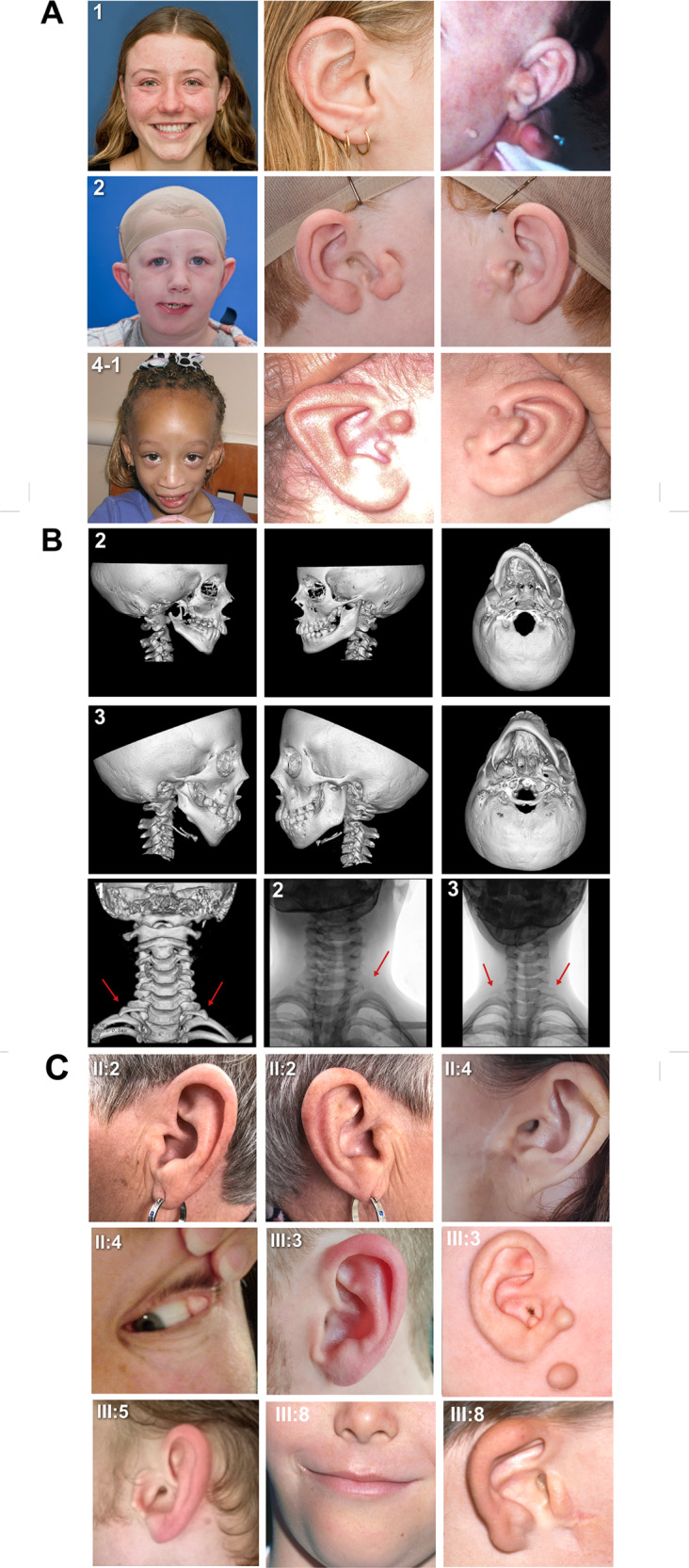
Clinical Images of Individuals with *SF3B2* haploinsufficiency. **A**. Photographs of three individuals demonstrating the shared phenotype. **Proband 1** (c.2329dupG) demonstrates mandibular hypoplasia on the left, scar from preauricular skin tag excision on the right, and tragal duplication and facial skin tag on the left side prior to removal. **Proband 2** (c.1780-2 A > -) demonstrates maxillary and mandibular hypoplasia on the right side in addition to bilateral tragal abnormalities. **Proband 4-1** (c. 1608dupG) presented with maxillary and mandibular hypoplasia and tragal abnormality on the left side, and bilateral preauricular tags. **B**. Three-dimensional (3D) computed tomography (CT) reconstructions of the craniofacial skeleton of probands 2 and 3. Frontal, lateral, and submental views demonstrate maxillary and mandibular hypoplasia with resulting facial asymmetry. The third row demonstrates, from left to right, 3D CT scans of the cervical spine of **Proband 1** and X-rays of **Proband 2 and 3**. Arrows in each figure indicate the cervical ribs. **C**. Photographs of family members with a shared *SF3B2* startloss variant (p.M1?) demonstrating variable external ear phenotypes. Images of **II:2** demonstrate a right ear markedly smaller than the left. Images of **II:4** demonstrate a tragal abnormality and scar from prior preauricular skin tag removal, in addition to an epibulbar dermoid. **III:3** presented with bilateral tragal abnormalities and tags (right side ear photo from childhood). Images of **III:5** show an underdeveloped tragus. **III:8** had tragal abnormality, scar from tag excision, right lateral oral cleft, and chin deviation due to mandibular hypoplasia.

To identify novel loci for CFM, we performed whole exome (*n* = 42) or whole genome (*n* = 104) sequencing on 138 case-parent trios with sporadic disease, and eight kindreds with multiple affected members. On average, we identified 1.11 coding de novo variants per proband, closely matching expectation and prior experimental results (Table 1)^12^. In comparing the burden of de novo variants in 138 trios with sporadic CFM to expectation, a significant excess of loss of function (LOF) variants was identified in cases (1.7-fold excess, *P* = 0.01). No other variant class demonstrated enrichment in comparison to expectation by chance (Table 1). A single gene, *SF3B2*(MIM: 605591), had more than one protein altering de novo variant, in which we identified de novo LOFs in two distinct probands: a single base deletion at a canonical splice acceptor (c.1780-2delA; Kindred 3) and a frameshift variant (p.D766EfsX4; Kindred 1). The identification of two de novo LOF variants in *SF3B2* in a cohort of this size was highly unlikely to occur by chance (*P* = 9.6 × 10^−7^; Supplementary Fig. 1), surpassing conservative thresholds for genome-wide significance. In a multiplex kindred, we identified a transmitted LOF in *SF3B2* (p.A827RfsX5; Kindred 2) in a proband with bilateral CFM, in whom the variant was inherited from an affected father (Fig. 1; Table 2). Strikingly, *SF3B2* lies within the 11q12-13 linkage region (odds in favor of linkage 2048:1) previously identified in the largest reported kindred demonstrating dominant CFM, a family included amongst our multiplex kindreds (Kindred 7)^10,11,13,14^. We performed whole exome sequencing of two distantly related individuals within this pedigree (cousins III:5 and III:8; Fig. 1). A single rare, damaging variant was shared between the two affected cousins within the linkage interval- a single nucleotide substitution causing a startloss in *SF3B2* (c.A1T; p.M1?). This variant segregated with 11 affected individuals, with phenotypes including ears with tragal abnormalities, isolated preauricular tags, maxillary and mandibular hypoplasia, epibulbar dermoids, and lateral oral clefts (Fig. 2C, Table 3). In total, we identified novel *SF3B2* variants in 4 kindreds. The burden of rare (gnomAD minor allele frequency < 2 × 10^−5^) LOF variants in *SF3B2* in this cohort was highly enriched in comparison to those amongst more than 250,000 alleles in gnomAD^15^ (*P* = 3.8×10^−10^; Fisher’s exact test), and each allele identified in CFM probands was novel (Table 4). *SF3B2* has a pLI score of 1.0, indicating very high intolerance to heterozygous LOF variation, and consequently a high likelihood of phenotypic consequence associated with its mutation. All four probands harboring these *SF3B2* variants presented with ear malformations limited to the tragus, mandibular hypoplasia, preauricular and/or facial tags, and cervical ribs in the three with radiographs available for study (Tables 2, 3; Fig. 2). Together, these findings implicate LOF variants in *SF3B2* as a cause of CFM.

**Table 1:** Burden of de novo variants in 138 probands with sporadic CFM.

	Observed		Expected		Enrichment	*P* value
Class	#	#/subject	#	#/subject		
All variants	153	1.11	154.2	1.12	0.99	0.55
Synonymous	48	0.35	43.8	0.32	1.10	0.28
Protein altering	101	0.73	110.4	0.80	0.92	0.83
Total missense	78	0.57	96.9	0.70	0.81	0.98
Damaging missense	15	0.11	18.2	0.13	0.83	0.80
Loss of function (LOF)	23	0.17	13.6	0.10	1.69	**0.01**

Bold values represent significant enrichment.

#, number of de novo variants in 138 probands with sporadic CFM; Rate, number of de novo variants per subject; Damaging missense as called by MetaSVM; Loss of function denotes premature termination, frameshift, or splice site variant. *P* values represent the uncorrected upper tail of the Poisson probability density function (one-sided).

**Table 2:** Clinical characteristics of individuals with pathogenic *SF3B2* variants.

Proband ID	1	2	3	4-1	4-2	5-1	5-2	6
Inheritance	de novo	dominant (pat)	de novo	Unknown^a^	Unknown^a^	de novo	de novo	Unknown^b^
Nucleotide change (GenBank NM_006842.2)	c.2329dupG	c.2480dupC	c.1780-2 A > -	c. 1608dupG	c. 1608dupG	c.1912 C > T	c.1912 C > T	c.307 C > T
Amino Acid change	p.D776EfsX4	p.A827RfsX5	–	p.R537AfsX24	p.R537AfsX24	p.R638X	p.R638X	p. Q103X
Gender	Female	Male	Male	Female	Male	Female	Male	Female
Age at exam (years)	17	9	10	12	17	30	28	1.5
Weight in Kg (%tile)	61 (50th −75th)	38 (90th–98th)	27 (10th–20th)	34 (25th)	82 (90th)	48 (3rd−10th)	71 (50th)	8 (3rd−10th)
Height in cm (%tile)	165 (50th −75th)	135 (50th–75th)	135 (20th–30th)	137 (<3rd)	156 (<3rd)	164 (50th)	178 (50th−75th)	67 («3rd)
OFC in cm (%tile)	54 (50th)	52 (20th–40th)	52 (20th–40th)	51 (3rd −10th)	50 (<3rd)	54 (25th−50th)	56 (50th)	42(«3rd)
Facial asymmetry	L mild mandibular hypoplasia	R moderate maxillary, mandibular, and zygomatic hypoplasia	R moderate maxillary and mandibular hypoplasia	L moderate maxillary and mandibular hypoplasia; L orbital dystopia	L mild maxillary and mandibular hypoplasia; L orbital dystopia	L maxillary and mandibular hypoplasia, zygomatic hypoplasia	–	–
Coloboma	–	–	–	–	–	–	–	Upper eyelid
Lateral oral cleft	–	–	R side	L side	–	Bilateral	–	–
Temporomandibular joint	–	Absence on R	–	–	–	–	–	–
Ear abnormalities	Duplication of tragus on L side	Duplication of tragus on R side	Bilateral hypoplastic tragus	Bilateral microtia I (EAC atresia on L)	Bilateral microtia I	Bilateral microtia I with R absent tragus, EAC atresia, and multiple sinuses	–	–
Skin Tags	L, facial	L, complex preauricular (2)	Bilateral, preauricular	L preauricular and facial (multiple)	–	–	L, complex preauricular (multiple)	R, preauricular (2)
Hearing (by audiologist test)	Normal	R, conductive HL	Normal	L, conductive HL	Normal	Bilateral conductive HL	Congenital perforation of L tympanic membrane	Normal
Ophthalmologic anomalies	Myopia	–	–	Ptosis, corrective lenses	Corrective lenses, amblyopia and strabismus	R epibulbar dermoid	–	R exotropia, hypermetropia, severe ptosis
Skeletal anomalies	Bilateral cervical ribs (C7)	Cervical rib on L (C7)	Bilateral cervical ribs (C7)	Scoliosis, Hypoplastic 12th ribs and non-rib bearing lumbar vertebral bodies (4), short toes	Bilateral extra flexion crease on thumbs, knee valgus, pes planus	–	–	L Bifid thumb
						(No X-ray)	(No X-ray)	(No X-ray)
Cardiac anomalies (Echocardiogram)	Absent L pulmonary artery, aberrant L subclavian artery, R sided aortic arch	NP	None	Multiple muscular VSD	None	NP	NP	NP
Kidney anomalies (US)	None	None	None	None	None	NP	NP	NP
Other birth defects	–	–	–	Submucous CP, Torticollis, anteriorly placed anus, dry skin, thin hair, scant eyebrows	Premature adrenarche, mild 2-3-4 skin syndactyly of digits	–	–	Mild skin syndactyly of toes
Neurodevelopment	Normal	Mild NDD (IEP)	Normal	NDD (IEP)	NDD (IEP)	Normal	Normal	Normal

*OFC* occipitofrontal circumference, *%tile* percentile, *R* right side, *L* left side, *NS* not specified, *HL* hearing loss, *VSD* ventricular septal defect, *US* ultrasound, *NDD* neurodevelopmental delay, *NP* not performed, *IEP* Individualized Education Program, *EAC* external auditory canal, *CP* cleft palate.

^a^Father not tested.

^b^Parents not tested.

**Table 3:** Clinical characteristics of individuals from Kindred 7, a family with autosomal dominant CFM and a pathogenic *SF3B2* variant (c.A1T:p.M1?).

Clinical features	I:2	II:2	II:4	II:7	II:9	III:1^a^	III:3	III:4	III:5	III:7	III:8	III:9
Gender	Female	Female	Female	Male	Male	Female	Male	Female	Male	Male	Male	Male
Age at last exam (years)	60	63	40	36	35	15	23	15	6	11	9	7
Facial asymmetry	L, mild mandibular hypoplasia	–	L, mild mandibular hypoplasia	–	R, mild mandibular hypoplasia	L, mild mandibular hypoplasia	R, mild maxillary and mandibular hypoplasia	–	R, mild mandibular hypoplasia	R, mild mandibular hypoplasia	R, moderate maxillary and mandibular hypoplasia	R, mild mandibular hypoplasia
Lateral oral cleft	–	–	–	–	R, mild	–	–	–	–	–	R, moderate	–
Temporomandibular joint^b^	–	–	L condylar hypoplasia	–	R condylar hypoplasia	–	R condylar hypoplasia	–	R condylar hypoplasia	R condylar hypoplasia	R condylar hypoplasia	R condylar hypoplasia
Ear abnormalities	–	R smaller than L	L abnormal tragus, absent lobule	R smaller than L	–	–	R abnormal tragus, absent lobule, EAC stenosis	–	R abnormal tragus, EAC stenosis	R abnormal tragus	R microtia I	–
Skin Tags	–	R preauric	L preauric	Bilateral preauric	R preauric	–	R preauric	R, preauric	R preauric	–	R preauric	Bilateral preauric
Hearing	Normal (NT)	Normal (NT)	Normal (NT)	Normal (NT)	Normal (NT)	Normal (NT)	Normal (NT)	Normal (NT)	Normal (NT)	Normal (NT)	Normal (NT)	Normal (NT)
Ophthalmological anomalies	–	–	L epibulbar dermoid	–	–	–	R epibulbar dermoid	–	–	–	–	–
Neurodevelopment	Normal	Normal	Normal	Normal	Normal	Normal	Normal	Normal	Normal	Normal	Normal	Normal
Other birth defects^c^	–	–	–	–	–	–	–	–	–	–	–	–

*NT* Not Tested, *R* right, *L* left, *EAC* external auditory canal, *preauric* preauricular.

^a^This individual was assessed as not-affected in the initial paper^11^; reassessment in 2020 revealed left mandibular hypoplasia.

^b^By evaluation of panoramic radiographs.

^c^Weight, height and head circumference were unremarkable (not formally documented). Cardiac, skeletal and kidney anomalies were not assessed with imaging.

**Table 4:** De novo and transmitted LOF variants in *SF3B2* identified in kindreds with CFM.

Gene	NM_006842.2 nucleotide change	Protein impact	gnomAD frequency	pLI
*SF3B2*	A1T	M1? (startloss)	Novel	1.00
*SF3B2*	C307T	Q103X	Novel	1.00
*SF3B2*	1608dupG	R537AfsX24	Novel	1.00
*SF3B2*	1780-2delA	IVS15-2delA	Novel	1.00
*SF3B2*	C1912T	R638X	Novel	1.00
*SF3B2*	2329dupG	D776EfsX4	Novel	1.00
*SF3B2*	2480dupC	A827RfsX5	Novel	1.00

*pLI* probability of LOF intolerance.

We assessed variant calls from probands for rare, damaging (as called by Meta-SVM) missense variants in *SF3B2*, as well as structural variants encompassing the gene, however none were identified. Analysis of structural variants in the DECIPHER database identified a child with a 1 Mb deletion encompassing *SF3B2* presenting with features of CFM (microtia, preauricular skin tag, other craniofacial dysmorphism)^16,17^, suggesting that deletions encompassing the gene could also cause some cases of CFM. No gene had more than one rare, recessive genotype, and no other single gene approached significance in analyzing the burden of rare, damaging alleles in cases. Excluding kindreds with *SF3B2* variants, analysis of de novo mutation burden in our cohort identified a persistently significant excess of de novo LOF mutations in cases (1.6-fold excess; *P* = 0.03, Poisson test; Supplementary Table 1). From the observed excess of de novo LOFs in cases compared to expectation, we estimate that an additional ~5.6% of cases are attributable to these mutations (Methods).

### Replication of haploinsufficient *SF3B2* variants in CFM

Using the Genematcher platform^18^, we identified an additional 5 individuals from 3 kindreds (Kindreds 4,5,6) with heterozygous LOF variants in *SF3B2* (p.R537AfsX34, p.R638X, p.Q103X). These individuals shared the CFM phenotype observed in our cohort, and some demonstrated novel cardiac and limb malformations (Table 2, Fig. 1). In sporadic cases wherein both parents were available for testing, these variants were found to occur de novo in the proband, including one kindred wherein two affected children shared a nonsense variant (p.R638X), however parental samples with confirmed kindship demonstrated no evidence of the variant, indicating gonadal mosaicism (Fig. 1, Table 2). Clinical features of each affected individual, Sanger sequencing traces of identified variants, and a clinical synopsis of each proband are found in Table 2, Supplementary Fig. 2, and Supplementary Note 1, respectively.

### SF3B2 knockdown interferes with neural crest development in *Xenopus laevis*

**Fig. 3:**
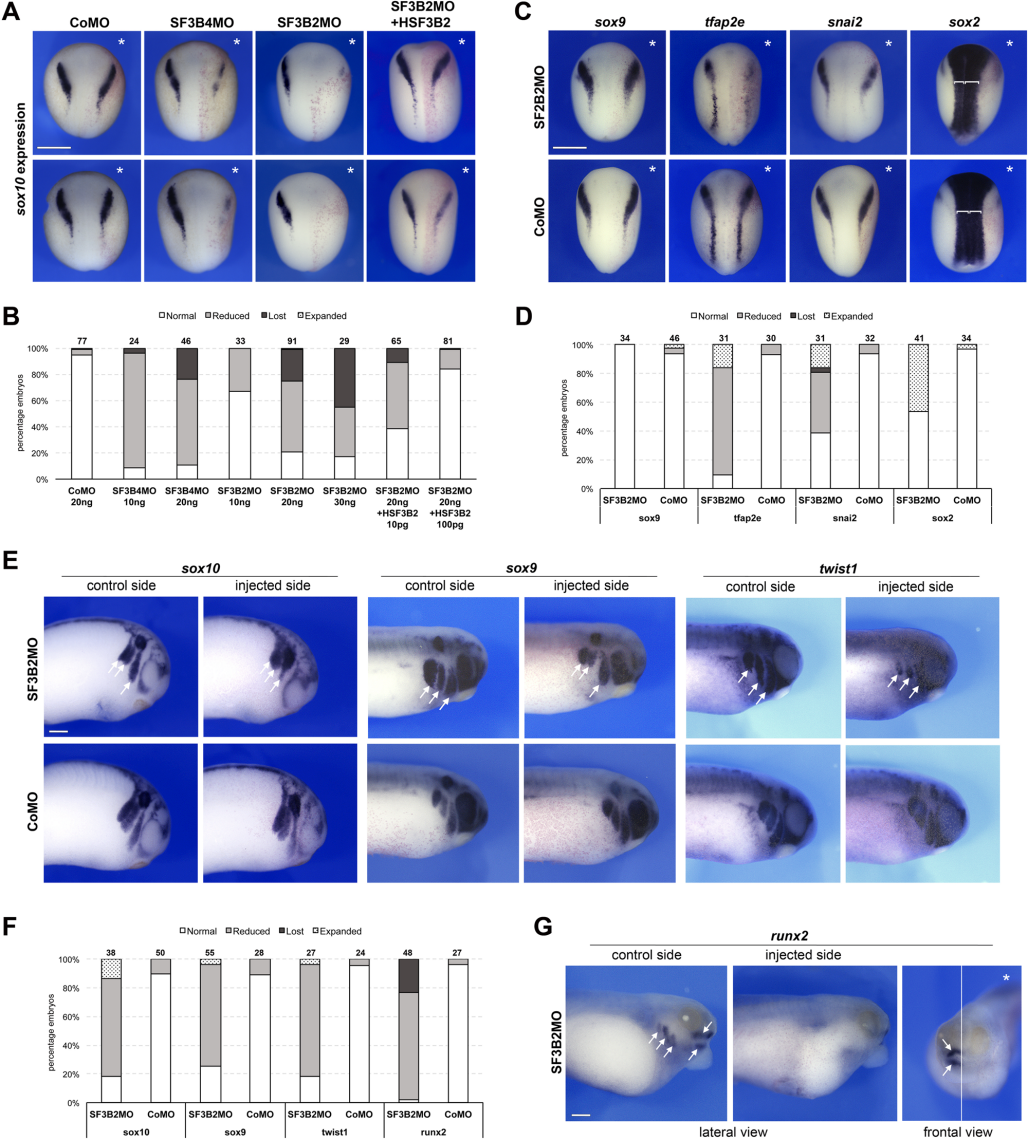
Sf3b2 knockdown alters neural crest development in *Xenopus* embryos. **A** Unilateral injection of increasing doses of SF3B2MO (10-30 ng) interferes with *sox10* expression at the neurula stage, in a manner similar to SF3B4 knockdown. Injection of a control MO (CoMO) did not significantly affect *sox10* expression. The SF3B2 knockdown phenotype is efficiently rescued by injection of human SF3B2 (HSF3B2; 10 pg or 100 pg) plasmid DNA. mRNA encoding the lineage tracer ß -galactosidase was co-injected with the MOs to identify the injected side (punctate red staining). The injected side is indicated by an asterisk. Dorsal view, anterior to top. The two rows show examples of the phenotype for each injection condition. Scale bar, 500 μm. **B** Quantification of the phenotypes. The numbers on the top of each bar indicate the number of embryos analyzed from seven independent experiments. **C** Unilateral injection of SF3B2MO (20 ng) reduces the expression of other neural crest genes at the neurula stage including *tfap2e* and *snai2*, and expanded the neural plate expression domain of *sox2* (white brackets) on the injected side. *sox9* was not affected in these embryos. The injected side is indicated by an asterisk. Dorsal view, anterior to top. Scale bar, 500 μm. **D** Quantification of the phenotypes. The numbers on the top of each bar indicate the number of embryos analyzed from three independent experiments. **E** At the tailbud stage, SF3B2MO (20 ng) injected embryos show a decrease in the length of the NC streams (arrows) as visualized by *sox10* (stage 25), *sox9*, and *twist1* (stage 28) expression. CoMO injection did not affect NC streams formation. Lateral view, dorsal to top, anterior to right. Scale bar, 250 μm. **F** Quantification of the phenotypes. The numbers on the top of each bar indicate the number of embryos analyzed from three independent experiments. **G** At stage 40, the expression of *runx2* in the pharyngeal arches mesenchyme (arrows) is severely downregulated in SF3B2MO-injected tadpoles. The white line indicates the midline (frontal view). Scale bar, 250 μm. The quantification of the *runx2* phenotype is shown in panel **F**. Source data are provided as a source data file.

**Fig. 4:**
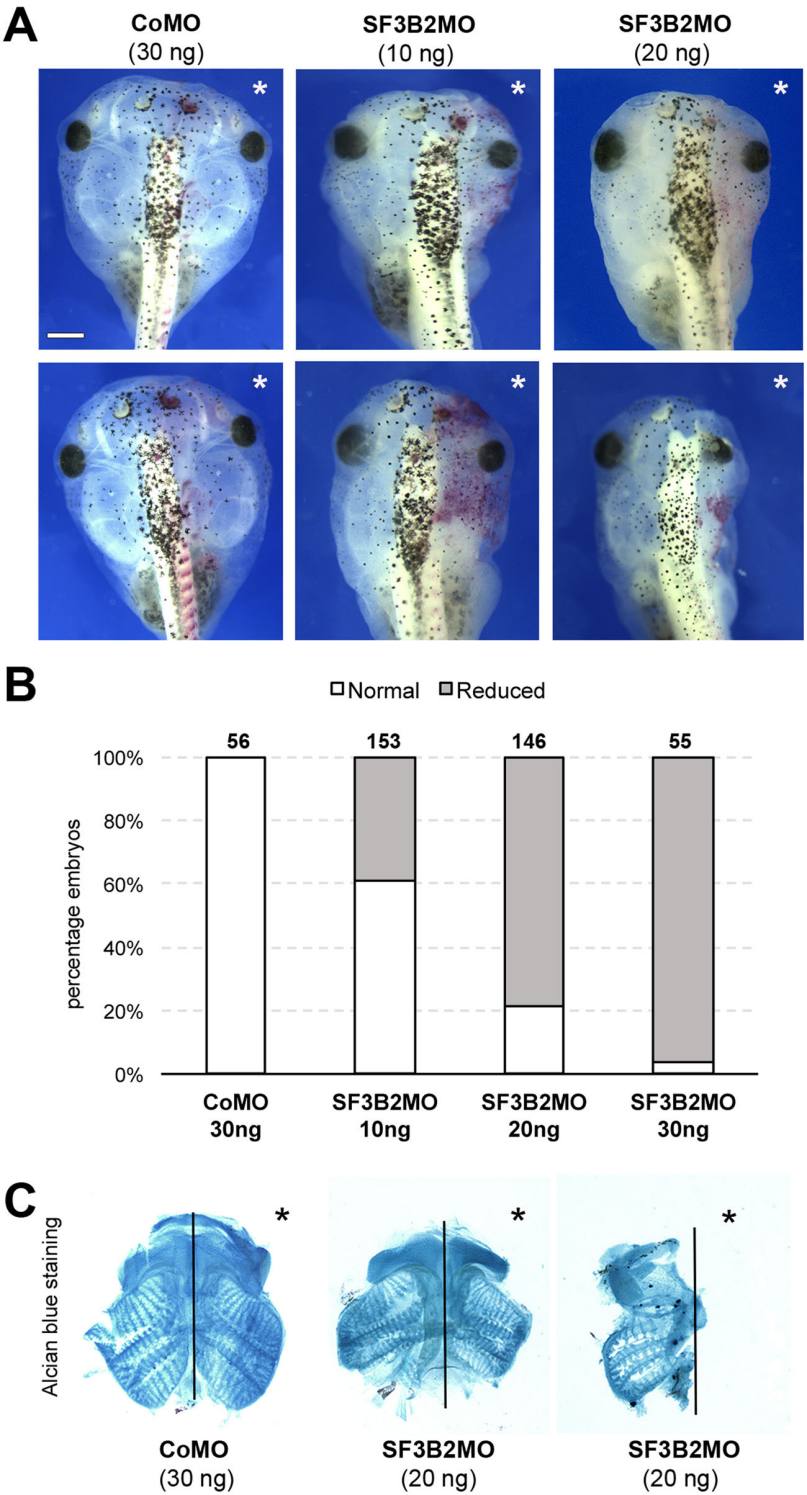
Sf3b2 knockdown causes craniofacial defects in *Xenopus* tadpoles. **A** Gross morphology of the head of SF3B2MO- and CoMO-injected *Xenopus* tadpoles. The lineage tracer ß -galactosidase was again co-injected, with the injected side indicted by an asterisk. Dorsal views, anterior to top. The two rows show examples of the phenotype for each injection condition. Scale bar, 600 μm. **B** The graph is a quantification of the results from three independent experiments. The number of tadpoles analyzed is indicated on the top of each bar. **C** Alcian blue staining of dissected craniofacial cartilages of CoMO (30 ng) and SF3B2MO (20 ng) injected tadpoles at stage 45. The injected side is indicated by an asterisk. The black lines indicate the midline. Source data are provided as a source data file.

The congenital anomalies seen in CFM suggest a disturbance in cranial neural crest (NC) cell formation, which typically migrate into the first and second pharyngeal arches. This paucity of NC cells is thought to underlie the craniofacial phenotype of CFM, particularly the underdevelopment of the mandible and external ear^19,20^. Variants in *SF3B4*, a binding partner of *SF3B2*, cause a craniofacial condition characterized by mandibular and external ear abnormalities known as Nager syndrome (MIM:154400)^21^. Previous work has shown that in an animal model of Nager syndrome, SF3B4 knockdown leads to loss of NC progenitors and subsequent defects in NC-derived craniofacial cartilages^22^. We sought to determine whether the craniofacial anomalies seen in probands with *SF3B2* haploinsufficiency might also be due to defects in NC precursor formation. We first confirmed the expression of *sf3b2* in *Xenopus* embryos by in situ hybrization where *sf3b2* appears to be ubiquitously expressed at all stages examined (Supplementary Figure 3). We then performed microinjection of *SF3B2* translation-blocking morpholino antisense oligonucleotides (SF3B2MO) in *Xenopus* embryos, and analyzed the expression of the NC specific gene *sox10* by in situ hybridization at the neurula stage (stage 15). Unilateral injection of increasing doses of SF3B2MO (10–30 ng) severely affected *sox10* expression in these embryos in a dose dependent manner, while injection of a control MO (CoMO) did not significantly affect the expression of this gene. For the highest dose of SF3B2MO injected (30 ng), 83% of embryos had a reduced or complete loss of *sox10* expression, identifying a dosage sensitive role of SF3B2 in NC progenitor formation (*P* < 0.005, One-way ANOVA using all injection concentrations (10–30ng); Fig. 3A, B). The phenotype of *SF3B2* morphant embryos was efficiently rescued by injection of human *SF3B2* plasmid DNA (10 and 100 pg) in a dose-dependent manner, demonstrating the specificity of the phenotype (Fig. 3A, B). We expanded our characterization of the morphant phenotype by assessing the expression of a broader repertoire of genes expressed in NC (*sox9, tfap2e, snai2*) or neural plate (*sox2*) progenitors. While *sox9* expression was largely unaffected, *tfap2e and snai2* were reduced, similar to *sox10*, and the *sox2* expression domain was expanded, consistent with a loss of neural plate border (Fig. 3C, D). At the tailbud stage (stages 25 and 28), when NC cells initiate their migration into the pharyngeal arches, SF3B2 morphant embryos showed a marked decrease in the dorso-ventral extension of the NC streams as visualized by *sox10*, *sox9* and *twist1* expression (Fig. 3E, F). At stage 40, *runx2* is detected in foci of cartilage precursors in the mesenchyme of the pharyngeal arches, and its expression in these domains was severly disrupted in SF3B2MO-injected tadpoles (Fig. 3F, G). We subsequently analyzed stage 45 *Xenopus* tadpoles to evaluate the long-term consequences of SF3B2 depletion on craniofacial development. For the lower doses of SF3B2MO (10 ng and 20 ng), we observed significant craniofacial defects affecting the overall size of the head on the injected side, while CoMO-injected tadpoles (30 ng) were unaffected (Fig. 4A, B). The phenotype was even more pronounced for the higher doses of SF3B2MO (30 ng), however only 35% of these embryos survived to the tadpole stage. Alcian blue staining revealed that NC-derived cartilages are hypoplastic or missing in SF3B2 morphant tadpoles (Fig. 4C). Altogether, these results indicate that the SF3B2 knockdown phenotype is similar to that observed in SF3B4 knockdown, and consistent with a depletion of NC progenitors contributing to the pathoetiology of CFM^22^.

## Discussion

In sum, we identified seven kindreds with LOF variants in *SF3B2*. These variants span the length of the encoded protein, supporting haploinsufficiency as the mechanism of genetic contribution to disease. Individuals with *SF3B2* variants showed phenotypic homogeneity via external ear malformations consistently involving the tragus, and mandibular hypoplasia. These findings suggest that these variants predominantly affect development of pharyngeal arch I, which gives rise to the mandible, ear canal, and the tragus^23^. Targeted knockdown of SF3B2 in *Xenopus* embryos indicates that in the absence of Sf3b2 function, the formation and subsequent development of cranial NC cells is disrupted, suggesting that NC depletion is a contributing factor to the pathoetiology of CFM. This is consistent with other craniofacial conditions, such as Treacher Collins and Nager syndromes^20,22^. Seventy-seven of the 146 kindreds in our cohort had phenotypes involving more than isolated microtia, and 4 of these harbored LOFs in *SF3B2*; we estimate that ~5% of CFM is attributable to variants in *SF3B2*. Notably, 25% of familial cases in our cohort were explained by *SF3B2* variants. Analysis of *SF3B2* variants in a larger cohort will be necessary to further specify the overall contribution of these mutations to CFM. Highly penetrant, dominant alleles have been found to cause a minor fraction (~5%) of the other congenital craniofacial anomalies that demonstrate similarly high locus heterogeneity, including *IRF6* in cleft lip/palate^24,25^, and *SMAD6* in craniosynostosis^12^. While there were no clear examples of incomplete penetrance in our cohort, one proband (Kindred 8; III:1) evaluated in infancy did not demonstrate features of CFM, however repeat clinical evaluation in adolescence after identification of the *SF3B2* variant revealed mandibular hypoplasia. These findings imply that those with *SF3B2* LOF mutations identified in infancy with no obvious manifestations of the condition should be assessed closely throughout their development for delayed, potentially mild presentations of CFM.

Lateral oral clefts and epibulbar dermoids, features observed in our probands, are two phenotypes that have classically been used to differentiate CFM from other mandibulofacial dysostoses^19^. The identification of widely variable phenotypes within single kindreds supports the notion that microtia and CFM are part of a single phenotypic spectrum. Further investigation will be necessary to determine the extent to which genetic, environmental, and stochastic factors contribute to the phenotypic heterogeneity observed with *SF3B2* haploinsufficiency.

Notably, the often unilateral nature of disease in CFM remains enigmatic. Other common craniofacial anomalies, including cleft lip and craniosynostosis, similarly present with unilateral disease more frequently than bilateral disease. Popular theories to explain this enigma include somatic “second hits” in genes wherein haploinsufficient germline variants predispose to disease, and genetic modifiers of phenotypic presentation. Large families demonstrating substantial phenotypic variability, much like the multiplex kindred presented above (Kindred 7), provide evidence against somatic events causing the disease phenotype in the case of *SF3B2*-associated CFM, as the recurrence of such events in a single gene across multiple generations would be highly unlikely. The potential contribution of somatic mutations in other genes, as well as genetic modifiers, merit further investigation in CFM and other congenital craniofacial anomalies.

The SF3B complex forms part of the U2 small nuclear ribonucleoprotein complex (U2 snRNP), binding to pre-mRNAs upstream of intron branch sites and anchoring the U2 snRNP to pre-mRNAs. SF3B2 directly interacts with SF3B4 to accomplish these functions. As described above, LOF variants in *SF3B4* cause the acrofacial dysostosis known as Nager syndrome^21^. Patients with Nager syndrome typically have symmetric disease involving the limbs, ears, midface, and mandible. Whereas external ear defects appear frequently in those with *SF3B2* variants, they are restricted to the tragal region and ear canal, and the malar and mandibular hypoplasia that are hallmark features of Nager syndrome appear to be less severe in probands with *SF3B2* variants. Limb malformations were infrequent in probands with *SF3B2*-associated CFM in contrast to the near complete penetrance of limb anomalies in Nager syndrome^21^. Whereas the majority of children with Nager syndrome require craniofacial surgery to maintain airway patency, none of the probands with *SF3B2* haploinsufficiency in this cohort required a tracheostomy or early mandibular surgery for airway compromise.

Interestingly, we identified cervical ribs in three individuals with *SF3B2* haploinsufficiency; the exact frequency in the cohort is unknown since this abnormality is commonly overlooked and we did not have radiographs available for review for the other individuals. Cervical ribs are found in ~1% of the general population, and are usually unilateral. Rib anomalies are frequent in patients with another craniofacial spliceosomopathy, cerebro-costo-mandibular syndrome, caused by variants in *SNRPB*^26^. These findings strengthen the link between spliceosome function and rib patterning in development.

Recent studies have elucidated a role of spliceosomal dysfunction in the retention of poison exons; these highly conserved alternative exons contain a premature termination codon, and are usually spliced out of pre-mRNAs. While found in many genes with significant roles in development and morphogenesis, the exact function of poison exons remains unknown^27^. We identify a significant loss of NC precursors in SF3B2 deficient embryos; it will be interesting to determine whether retention of poison exons via aberrant splicing might contribute to this disease process.

The results define *SF3B2* as a novel gene responsible for CFM (Supplementary Fig. 4), demonstrating dominant inheritance with inter- and intrafamilial phenotypic variability. We recommend adding *SF3B2* to craniofacial genetic panels designed to screen patients with features of mandibulofacial dysostosis. Genetic testing will be particularly useful for CFM patients with positive family history, tragal malformations, or preauricular/facial tags, as the high penetrance of these alleles has strong implications for genetic counseling. Recruiting a larger cohort of patients with *SF3B2* variants will be necessary to delineate the full spectrum of phenotypic consequences associated with its mutation. Given the apparently high degree of locus heterogeneity in CFM, the results suggest that sequencing a substantially larger cohort of CFM cases is likely to reveal other genes and pathways in which de novo mutations contribute to disease risk.

## Methods

### Sample population

To explore the genetic etiology of CFM, we enrolled families with CFM in our multinational research consortium from 2009 to 2020. Individuals between 0 and 18 years of age were eligible for the study if they met at least one of the following inclusion criteria: (1) microtia or anotia; (2) mandibular hypoplasia and preauricular tag; (3) mandibular hypoplasia and facial tag; (4) mandibular hypoplasia and epibulbar dermoid; (5) mandibular hypoplasia and lateral oral cleft (e.g. macrostomia); (6) preauricular tag and epibulbar dermoid; (7) preauricular tag and lateral oral cleft; (8) facial tag and epibulbar dermoid; (9) lateral oral cleft and epibulbar dermoid; and (10) consenting parent spoke a language in which they were eligible for consent at their enrolling site. Individuals were excluded if they had an abnormal karyotype or a syndromic diagnosis that involves microtia or underdevelopment of the jaw. Detailed phenotype was ascertained from physical examination performed by a medical geneticist or medical chart abstraction, standardized 2D photos, and parental interview on medical history. Prenatal and family history was collected to account for exposure to known teratogenic substances, and to assess recurrence of the phenotype in the family. Blood or saliva samples were collected from the proband and available parents and affected relatives. DNA was extracted from blood and saliva using standard procedures (Qiagen and Oragene kits). Replication cases were obtained using the GeneMatcher (https://genematcher.org/) or DECIPHER (https://decipher.sanger.ac.uk/) platform.

All human studies were reviewed and approved by the institutional review board (IRB) of the Seattle Children’s Hospital and at each of the local institutions’ IRBs. An informed consent form was signed by all participants, and explicit written consent was provided for publication of clinical images of the face, which are identifiable. The authors affirm that human research participants have seen and read the material to be published and have provided informed consent for publication of the images in Fig. 2.

### Exome and genome sequencing

Exome sequencing was performed at either the Northwest Genomics Center (University of Washington) or GENEWIZ using the Nimblegen SeqCap Human Exome v2.0 and Agilent SureSelect Human All Exon capture platforms respectively. For three samples studied at GeneDx, exons were captured using the SureSelect Human All Exon V4 kit, the Clinical Research Exome kit, or the IDT xGen Exome Research Panel v1.0. Whole genome sequencing was performed at the Broad Institute or the Northwest Genomics Center, with sequence data generated on the Illumina HiSeq X platform.

### Variant analyses

Variants were called using the GATK pipeline (https://gatk.broadinstitute.org/) and annotated using ANNOVAR (http://www.annovar.openbioinformatics.org/), with allele frequencies derived from the gnomAD database (https://gnomad.broadinstitute.org/)^15,28^. De novo variants were called using TrioDeNovo (https://genome.sph.umich.edu/wiki/Triodenovo)^29^. Variants contributing to significant results were validated by bidirectional Sanger sequencing of the proband and both parents. The impact of nonsynonymous variants was predicted using the MetaSVM rank score^30^. Analysis of de novo variant burden in cases was performed in R using denovolyzeR (denovolyzer.org), wherein the observed distribution of variants was compared to the expected Poisson distribution^31,32^. To assess the burden of LOF variants in 145 kindreds with CFM, the total number of LOF alleles identified in each gene in cases was compared to the number identified amongst the median number of alleles across all variant positions (250,396 alleles for *SF3B2*) in gnomAD using Fisher’s exact test.

### Contribution of de novo mutations to CFM

We infer that the number of probands with de novo LOF mutations amongst 136 trios with sporadic CFM without *SF3B2* mutations (*n* = 21) in excess of those expected by chance (*n* = 13.4) represents the number of subjects in whom these mutations confer CFM risk (*n* = 7.6). Comparing this number to the total number of trios considered (7.6/136) yields the fraction of patients in whom these mutations are expected to contribute to disease risk: ~5.6%.

### Morpholino analysis in *Xenopus laevis*

*Xenopus laevis* embryos were raised in 0.1X NAM (Normal Amphibian Medium)^33^. The coding sequence of human SF3B2 (HSF3B2) inserted into pCS2+ expression vector was purchased from GenScript (Piscataway, NJ). Morpholino antisense oligonucleotides (MOs) were purchased from GeneTools (Philomath, OR). Control (CoMO), SF3B4 (SF3B4MO, GCCATAACCTGTGAGGAAAAAGAGC)^22^, and two SF3B2 translation blocking MOs (SF3B2MO) targeting each *sf3b2* allo-allele (SF3B2.SMO, CATATCCTCTCTACTTCCATATAAA; SF3B2.LMO, TTCCGCCATGTTTGCGGTATTTAAG; used at a 1:1 ratio) were injected at the 2-cell stage in one blastomere, along with 500 pg of ß-galactosidase mRNA as a lineage tracer to identify the injected side, and the embryos cultured until stage 15, stage 25, stage 28 or stage 40. For rescue experiments SF3B2MO (20 ng) was coinjected with 10 pg or 100 pg of pCS2 + HSF3B4 plasmid DNA. Prior to in situ hybridization, embryos were fixed in MEMFA (0.1 M MOPS, 2 mM EGTA, 1 mM MgSO_4_ and 3.7% formaldehyde) and stained for Red-Gal (Research Organics; Cleveland, OH) to visualize the lineage tracer (ß-gal mRNA). Antisense digoxygenin-labeled probes (Genius Kit; Roche, Indianapolis, IN) were synthesized using template cDNA encoding *sox10, sox9, snai2, tfap2e, twist1, runx2 and sox2*^34–40^. Whole-mount in situ hybridization was performed^41^. For Alcain blue staining, stage 45 tadpoles were fixed in MEMFA for 1 h, rinsed in H_2_O, skinned, eviscerated and stained^22^. Oligonucleotides used are described in Supplementary Table 2. All experiments performed were approved by the NYU Institutional Review Board and Institutional Animal Care and Use Committee.

## Supplementary information

Supplementary Information

Peer Review File

## Data Availability

The genetic sequencing data generated in this study have been deposited in the dbGaP database under accession code phs002130.v1.p1. Source data are provided with this paper.

## Source data

Source Data
