# Supplementary material for: Haploinsufficiency of SF3B2 causes craniofacial microsomia
Source: Nat Commun. 2021 Aug 3;12:4680. doi: 10.1038/s41467-021-24852-9 (PMC8333351; doi:10.1038/s41467-021-24852-9)
Supplement: Supplementary file 1 — Supplementary Information [file 41467_2021_24852_MOESM1_ESM.pdf]

Haploinsufficiency of *SF3B2* causes craniofacial microsomia

Supplemental Information

Table of Contents:

Supplementary Table 1. Burden of *de novo* variants in 136 probands with sporadic CFM without *SF3B2* variants.
Supplementary Table 2. Oligonucleotide sequences.

Supplementary Figure 1. Quantile-quantile plot of log transformed P values for enrichment of *de novo* LOF variants in each human gene.
Supplementary Figure 2. Sanger sequencing traces of *SF3B2* variants identified in kindreds with CFM.
Supplementary Figure 3. Developmental expression of *sf3b2* in Xenopus embryos.
Supplementary Figure 4. Spliceosome genes associated with craniofacial disease.

Supplemental Note 1: Clinical synopses of probands with haploinsufficient *SF3B2* variants.
Supplemental Note 2: University of Washington Center for Mendelian Genomics

Supplementary Table 1. Burden of *de novo* variants in 136 probands with sporadic CFM without *SF3B2* variants

	Observed		Expected		Enrichment	P-value
Class	#	#/subject	#	#/subject		
All variants	151	1.11	152.0	1.12	0.99	0.61
Synonymous	48	0.35	43.1	0.32	1.11	0.25
Protein altering	99	0.73	108.9	0.80	0.91	0.84
Total missense	78	0.57	95.5	0.70	0.82	0.97
Damaging missense	15	0.11	17.9	0.13	0.84	0.79
Loss of function (LOF)	21	0.15	13.4	0.10	1.57	**0.03**

#, number of *de novo* variants in 136 probands with sporadic CFM, exclusive of 2 sporadic cases found to have *de novo* variants in *SF3B2*; Rate, number of *de novo* variants per subject; Damaging missense as called by MetaSVM; Loss of function denotes premature termination, frameshift, or splice site variant. P-values represent the upper tail of the Poisson probability density function.

Supplementary Table 2. Oligonucleotide sequences

Morpholino	Oligonucleotide Name	Sequence
sf3b4 morpholino	SF3B4MO	GCCATAACCTGTGAGGAAAAAGAGC
sf3b2 morpholino allo-allele 1	SF3B2.SMO	CATATCCTCTCTACTTCCATATAAA
sf3b2 morpholino allo-allele 2	SF3B2.LMO	TTCCGCCATGTTTGCGGTATTTAAG

Supplementary Figure 1.

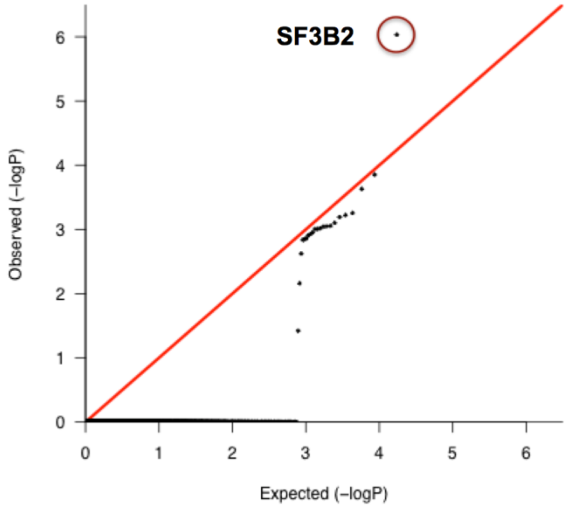

Supplementary Figure 1. Quantile-quantile plot of log transformed P values for enrichment of *de novo* LOF variants in each human gene.
The observed results match the expected distribution with the exception of *SF3B2*, which demonstrates significant enrichment ($P=9.6 \times 10^{-7}$; Poisson distribution). Source data are provided as a Source Data file.

Supplementary Figure 2.

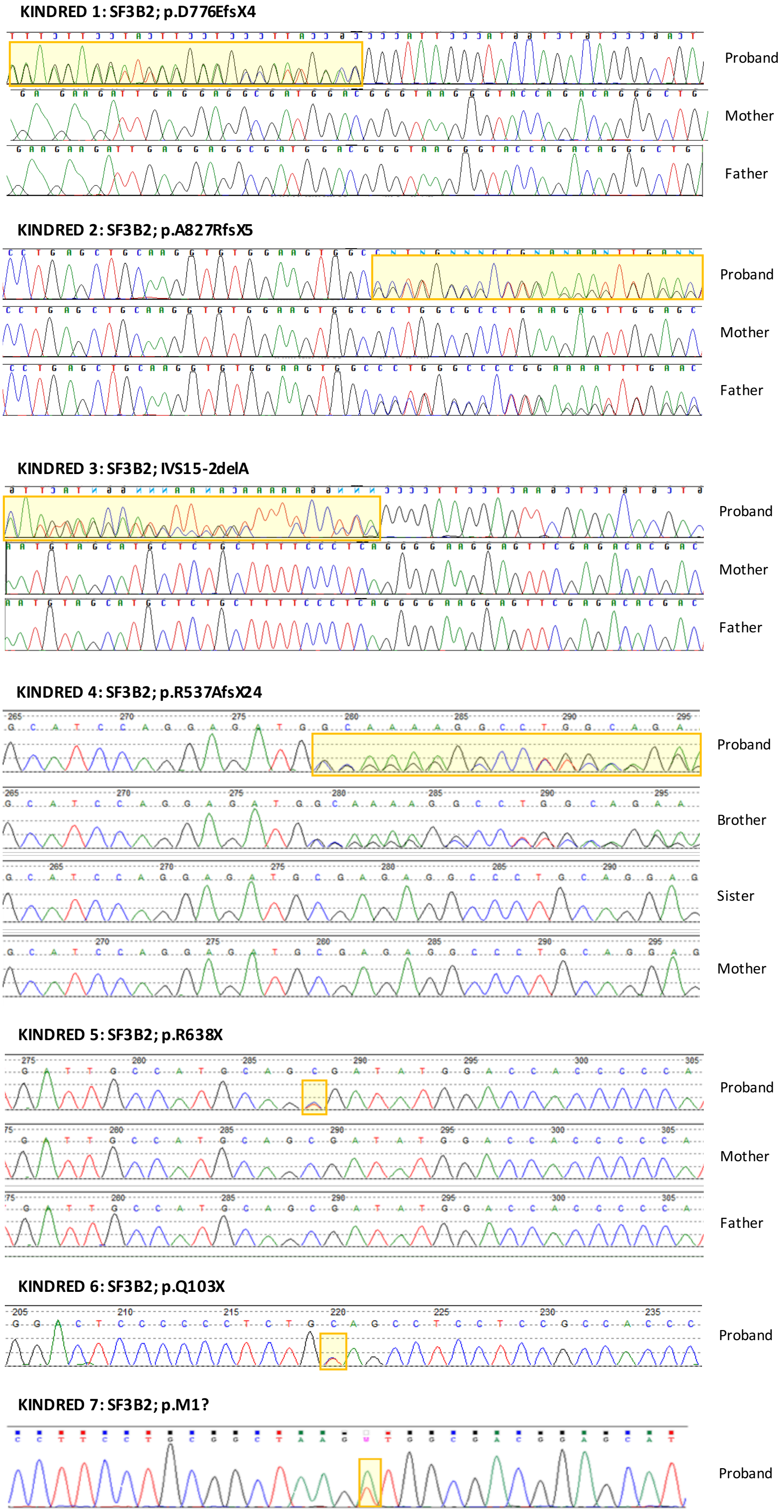

Supplementary Figure 2. Sanger sequencing traces of *SF3B2* variants identified in kindreds with CFM. Traces demonstrate the putative variant indicated in each pedigree described in the text. Highlighted bases demonstrate the variant indicated above each set of traces.

Supplementary Figure 3.

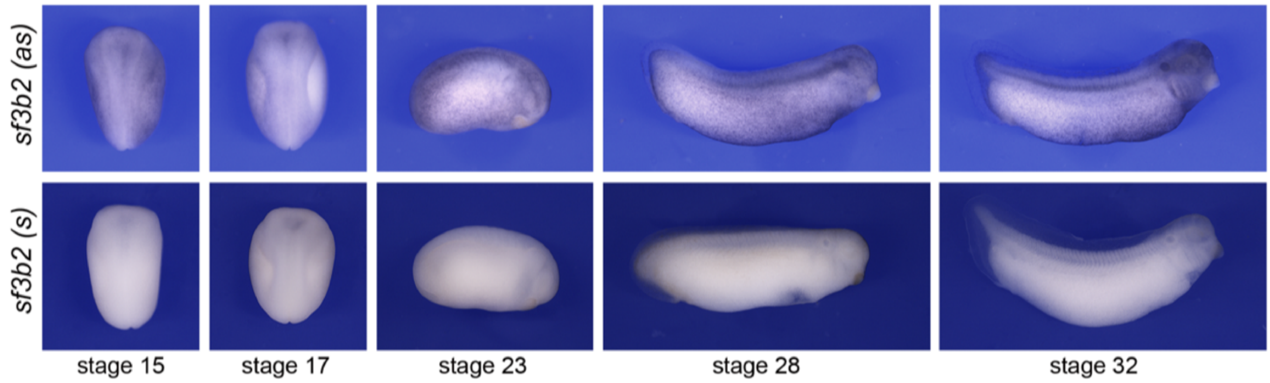

Supplementary Figure 3. Developmental expression of *sf3b2* in *Xenopus* embryos. By *in situ* hybridization, at all stages examined, *sf3b2*(as) appears to be ubiquitously expressed. Sense probe, *sf3b2*(s), is shown as control. Stages 15/17 are dorsal views, anterior to top. Stages 23/28/32 are lateral views, dorsal to top, anterior to right.

Supplementary Figure 4.

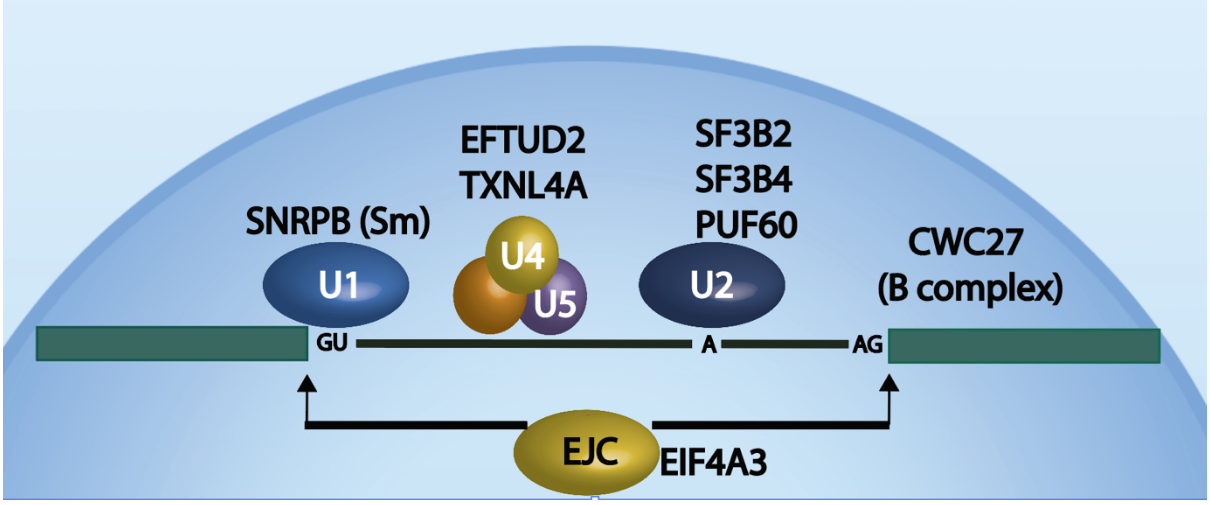

Supplementary Figure 4. Spliceosome genes associated with craniofacial disease. EJC; exon junction complex. SF3B2 is a component of the U2 snRNP, which participates in branch site recognition. Not pictured is RNU4ATAC, a disease gene which is a part of the minor U12-spliceosome.

Supplemental Note 1

Clinical synopses of probands with haploinsufficient *SF3B2* variants.

- ***KINDRED 1***

The proband is a 17-year-old white female delivered to a 29-year-old G1P1 mother after an uncomplicated pregnancy. She was delivered vaginally at 41 weeks gestational age. At birth, she was noted to have craniofacial anomalies including duplication of the tragus (preauricular tags) and a facial tag (cheek) on the left side, and mild left mandibular hypoplasia. Her birth weight was 3.1kg; birth length and head circumference are unknown. She went home shortly after birth.

At three years of age, a cardiac catheterization and an echocardiogram showed an absent left pulmonary artery with an aberrant left subclavian artery and a right-sided aortic arch. She also has pulmonary hypoplasia on the left side secondary to the abnormal vasculature.

Her development and growth are within normal limits. She was doing well at school (High school) during her last clinical visit. She has had a normal renal ultrasound and a normal hearing evaluation. She has myopia. A CT of the cervical spine revealed bilateral cervical ribs.

Family history is negative for birth defects or consanguinity.

Whole genome sequencing of the proband and both parents identified a *de novo* frameshift variant (p.D776EfsX4). The result was confirmed by Sanger sequencing (Supplementary Figure 2).

- ***KINDRED 2***

The proband is a 14-year-old white male delivered vaginally at term to a 31-year-old G1P1 mother after an uncomplicated pregnancy. At birth, he was noted to have craniofacial anomalies including bilateral preauricular tags and ear canal stenosis on the right side. His birth weight was 3 kg; birth length and head circumference are unknown. He went home shortly after birth.

The hearing evaluation showed a normal hearing on left side and a mild to moderate conductive hearing loss in the right ear. His cervical spine x-rays showed a small cervical rib on the left. The CT of the head showed hypoplasia of the right hemimandible including the mandibular condyle, hypoplasia of the right maxilla, and incomplete formation of the right zygomatic arch. Thinning of the soft tissues with contour deformity contiguous with the anteriorly displaced anterior portion of the right external ear was also evident. The right external auditory canal is minimally narrowed throughout. Limited evaluation of the ossicles and inner ear structures were unremarkable. He has had a normal renal ultrasound.

At three years of age he presented speech delay and was diagnosed with a moderate motor speech disorder. At nine years of age, he was in 2nd grade and receiving speech therapy and an Individualized Education Program. His growth is within normal limits.

His family history is significant for having his father with a left ear tag and his mother with bilateral ear tags. He has a healthy sister. Parents are non-consanguineous.

Whole genome sequencing of the proband and both parents identified a paternally inherited frameshift variant (A827RfsX5). The result was confirmed by Sanger sequencing (Supplementary Figure 2).

- ***KINDRED 3***

The proband is a 10-year-old Asian-Indian male delivered to a 32-year-old G1P0 mother after an uncomplicated pregnancy. He was delivered at 39 weeks gestational age via emergency cesarean section for breech presentation and maternal hypertension. At birth, he was noted to have craniofacial anomalies including bilateral preauricular tags, right lateral oral cleft, and right mandibular and maxillary hypoplasia. His birth weight was 3kg; birth length and head circumference are unknown. He had some difficulty feeding as a newborn, related to his lateral oral cleft, and physiologic newborn jaundice. He was otherwise well and went home shortly after birth.

His development and growth are within normal limits. He was doing well at school (5th grade) during his last clinical visit. He has had normal echocardiography, renal ultrasound, cervical spine x-rays, hearing and vision evaluations. A CT of the head revealed bilateral cervical ribs and confirmed the hypoplastic right hemi-mandible.

Family history is negative for birth defects or consanguinity.

Exome sequencing of the proband and both parents identified a *de novo* single base deletion within a canonical splice acceptor in *SF3B2* (IVS15-2delA). The result was confirmed by Sanger sequencing (Supplementary Figure 2).

- ***KINDRED 4-1***

The proband is a 14-year-old black female, followed since age 2 months for craniofacial microsomia and developmental concerns. She was delivered at 39 weeks gestation to a 30-year-old G2P1 mother after a pregnancy complicated by gestational diabetes and preterm labor. Birth weight was 2.9 kg, length was 48 cm, and head circumference was 33.5 cm. She was noted to have facial asymmetry with multiple preauricular and facial skin tags. She had bilateral anomalous ears and failed her newborn hearing screen on the left side, correlating with a stenotic left ear canal. She had multiple mid muscular ventricular septal defects. A renal ultrasound was normal. She had early feeding problems and failure to thrive, requiring nasogastric supplementation.

At two months of age, she was noted to have submucous cleft palate, light colored scalp hair with dry texture, moderate left hemifacial microsomia, a dimple on the left cheek lateral to the mouth with left lateral oral cleft, prominent sublingual frenulum, torticollis, wide spaced nipples and a narrow thorax, anteriorly placed anus, mild hypertonicity, brisk patellar reflexes, and a high-pitched, weak cry.

At age 12 when she was last seen, she was still petite in stature and was also noted to have small, broad, proximally placed first toe and ectodermal variations including poor hair growth, dry skin, poor dentition and generalized mild hypopigmentation compared to other family members. She had had multiple craniofacial surgeries. She was diagnosed with mild orbital dystopia and temporal bone, zygoma, maxilla and mandible hypoplasia. Her muscular VSDs had closed spontaneously. She was in the 7th grade at school with an IEP in place; she was reported to be working at a 3rd grade level. She was followed closely by neurology for ADHD and behavioral problems. Neurologic exam was normal. She had a delayed bone age (10 years at a chronologic age of 12 years and 6 months) and spine films showed hypoplastic 12th ribs and 4 non-rib bearing lumbar vertebral bodies.

The family history is notable for the mother having significant learning disabilities and insulin dependent diabetes. A chromosome microarray of the mother showed a microdeletion of 18p11.22, not present in any of her children. The mother's monozygotic twin sister reportedly has significant cognitive impairment. The proband's father is reported to be 5'2" and has a history of learning disabilities. His craniofacial features are unknown. There is a younger maternal half-sister with unilateral preauricular tag. The patient's brother is described separately.

Exome sequencing of the proband, her mother, her full brother and maternal half-sister identified a heterozygous frameshift variant in *SF3B2* (p.R537AfsX24) only in the proband and her full brother. The result was confirmed by Sanger sequencing (Supplementary Figure 2). The proband also has a microduplication of chromosome 2q13, arr[GRCh37]2q13 (111427222_113191231)X3. The duplication is not present in the patient's mother, brother or half-sister.

- ***KINDRED 4-2***

The proband is a 17-year-old black male. He was born to a 26-year-old G1P0 mother whose pregnancy was complicated by maternal hypertension and gestational diabetes. He was delivered at term by C-section due to fetal bradycardia; birth weight was 4.3kg. He had mild neonatal jaundice, but no neonatal complications.

At age four, he was noted to have microcephaly, amblyopia and strabismus, mild facial asymmetry with mild left maxillary and mandibular hypoplasia. His ears were notably small (but normally formed). He also had hypotonia, mild hyporeflexia, poor coordination, mild 2-3-4 syndactyly, extra flexion crease on each thumb, knee valgus and pes planus. He did not develop a social smile until he was five months old and did not walk until he was two years old. At age four, he was still using incomplete phrases and sentences. At age six, he had a brain MRI scan that showed no abnormalities.

His height and head circumference were consistently at around the 3rd percentile. He had pubic hair and axillary odor detected at approximately age 9, but normal bone age and endocrine screening. He complained of muscle aches and was recently noted to have tachycardia and mildly increased blood pressure. ECG showed sinus tachycardia, possible left axis deviation with T wave changes, but an echocardiogram was normal. He was diagnosed with ADHD

and oppositional defiance disorder and received behavioral counseling, occupational and speech therapy. At 17, he is in a special education 11th grade glass with an IEP.

Family history is described above on his sister's clinical summary (Individual 4-1).

He was found to be heterozygous for the same frameshift variant in *SF3B2* as his sister (p.R537AfsX24).

- ***KINDRED 5-1***

The proband is a 30-year-old white G2P1L1 female. She was born to a 28-year-old G2P1L1 mother and a 27-year-old father. The parents' first pregnancy was 4 years earlier and resulted in a son who is healthy. The parents were healthy and non-consanguineous. The pregnancy with the proband was uncomplicated and there was no history of skin rash, fever, high blood pressure, diabetes or exposure to teratogens. There were no abnormalities identified on fetal ultrasounds and screening for Down syndrome and open neural tube defects were not performed. Deliver was at 39 + 2/7 weeks gestation and was vaginal and uncomplicated. At birth she was noted to have craniofacial anomalies including a right conjunctival dermoid in the lateral canthus, macrostomia caused by bilateral transverse facial clefts, more severe on the left side, the right auricle was very small and had deficiency of the lower third of the ear, as well as obliteration of the external auditory meatus with an absent tragus and multiple sinuses. The left auricle was larger than the right with absence of the lower third and obliteration of the external auditory meatus. There was a sinus in the area of the obliterated external auditory meatus which discharged fluid. The left maxilla and mandible were hypoplastic. There were no other abnormalities, and the palate was intact. There was bilateral conductive hearing loss.

CT 3D complex head showed bilateral hypoplasia more severe on the left with the left mandibular ramus and mandibular condyle being smaller than the right, and the maxilla and zygomatic arch were also smaller. The pinna were hypoplastic bilaterally with absent auditory canal bilaterally. On the right the ossicular chain appeared to be fixated towards the scutum. Her development and growth were within normal limits.

Exome sequencing of the proband and both parents identified a *de novo* variant (c.1912C>T)(p.R638X) in *SF3B2*. The result was confirmed by Sanger sequencing (Supplementary Figure 2).

- ***KINDRED 5-2***

Her brother was born 2 years later and is currently 28 years old. The pregnancy with him was uncomplicated and there was no history of skin rash, fever, high blood pressure, diabetes or exposure to teratogens. There were no abnormalities identified on fetal ultrasounds and screening for Down syndrome and open neural tube defects were not performed. Delivery was at 39 weeks gestation and was vaginal and uncomplicated. At birth, he was noted to have left preauricular skin and cartilaginous tags in front and internal to the tragus. He was later found to have congenital perforation of the left tympanic membrane. No other abnormalities were noted and there was no facial asymmetry. His growth and development have been normal.

Exome sequencing of the proband and both parents identified a *de novo* variant (c.1912 C>T)(p.R638X) in *SF3B2*; this was the same variant identified in his sibling who also had CFM, demonstrating gonadal mosaicism. The result was confirmed by Sanger sequencing (Supplementary Figure 2).

- ***KINDRED 6***

The proband is a white female assessed in our clinic at the age of 18 months. She was born to a 26-year-old primigravida woman with poorly controlled diabetes mellitus type 2, essential hypertension, obesity, ADHD and anger control issues. The mother was on nifedipine, metformin and insulin during the pregnancy, but her glycemia was poorly controlled. She also smoked during the pregnancy. Prenatal ultrasounds were concerning for the fetus being small for gestational age. Delivery was induced at 36 weeks of gestational age for maternal hypertension. No resuscitation was required at birth. Birth weight was 2.4kg.

Preauricular skin tags and ptosis on the right and bifid distal thumb phalanx on the left were noted at birth. She had also significant exotropia and hypermetropia on the right. Ptosis was surgically

corrected at the age of 13 months and exotropia at 3 years of age. There have been no concerns about hearing. Since birth she has been growing steadily below the 3rd percentile curve. Development was appropriate for age at 18 months.

Chromosomal microarray revealed 10p11.22 microdeletion, a variant of unknown clinical significance, inherited from her healthy father. Exome sequencing on the proband revealed a nonsense variant in *SF3B2* (p.Q103X). This variant was confirmed by Sanger sequencing (Supplementary Figure 2).

- ***KINDRED 7 (first reported in Singer et al, 1994)***

The index case (**III:8**) was a 9-year-old (at the time of first examination), white, male. At birth he was noted to have right sided microtia and a preauricular skin tag. In addition, he had a right lateral oral cleft that was surgically repaired shortly after birth with simultaneous excision of his right preauricular skin tag. His growth and development were normal.

There was a strong family history spanning 3 generations of facial asymmetry, ear anomalies, preauricular skin tags, mandibular ramus and condyle abnormalities, and epibulbar dermoids, with variable penetrance amongst affected family members.

The family pedigree was updated from the previous publication as follows, individual **I:2** had facial asymmetry when assessed by the clinical team via photographic images. Individual **III:1** was assessed to have no manifestations in infancy, but recent reassessment shows left sided facial asymmetry and mandibular hypoplasia that became apparent in teenage years. **II:4** in the original report[1] was incorrectly assigned as affected but is, in fact, unaffected. **III:3** was born after the initial publication. At birth, he was found to have mandibular hypoplasia, 2 preauricular tags, an absent tragus, and a lateral epibulbar dermoid on the right side. He reports Eustachian tube dysfunction on the right side as well. Additionally, he has absent facial hair growth on his right side overlying the mandibular ramus region. **III:4** was also born after the 1994 published report. Her only manifestation of CFM is a right sided preauricular skin tag.

Supplemental Note 2.

University of Washington Center for Mendelian Genomics

Michael J. Bamshad[1,2], Suzanne M. Leal[3], and Deborah A. Nickerson[1]
Peter Anderson[1], Tamara J. Bacus[1], Elizabeth E. Blue[1], Kati J. Buckingham[1], Jessica X. Chong[1], Diana Cornejo Sánchez[3], Colleen P. Davis[1], Christian D. Frazar[1], Danielle Giroux[1], William W. Gordon[1], Martha Horike-Pyne[1], Jameson R. Hurless[1], Gail P. Jarvik[1], Eric Johanson[1], J. Thomas Kolar[1], Melissa P. MacMillan[1], Colby T. Marvin[1], Sean McGee[1], Daniel J. McGoldrick[1], Betselote Mekonnen[1], Patrick M. Nielsen[1], Karynne Patterson[1], Benjamin Pelle[1], Aparna Radhakrishnan[1], Matthew A. Richardson[1], Gwendolin T. Roote[1], Erica L. Ryke[1], Isabelle Schrauwen[3], Kathryn M. Shively[1], Joshua D. Smith[1], Monica Tackett[1], Machiko S. Threlkeld[1], Gao Wang[3], Jeffrey M. Weiss[1], Marsha M. Wheeler[1], Qian Yi[1], Jordan E. Zeiger[1], and Xiaohong Zhang[1]

[1] University of Washington
[2] Seattle Children's Hospital
[3] Columbia University

Supplementary References

1. Singer SL, Haan E, Slee J, Goldblatt J. Familial hemifacial macrosomia due to autosomal dominant inheritance. Case reports. Australian Dental Journal 1994; 39(5):287-291.
